# Fibrin Monomer and Systemic Lupus Erythematosus Reactivation During Pregnancy: A Retrospective Study

**DOI:** 10.3390/diseases13070210

**Published:** 2025-07-03

**Authors:** Tran Thi Kieu My, Hoang Thi Ha, Nguyen Huu Truong, Dao Thi Thiet, Nguyen Khanh Ha, Tran Dang Xoay, Linus Olson, Bach Quoc Khanh

**Affiliations:** 1 Division of Hematology, Hanoi Medical University, Hanoi 100000, Vietnam; 2 National Institute of Hematology and Blood Transfusion, Hanoi 100000, Vietnam; daothiet.nihbt@gmail.com (D.T.T.); ha.ngnkhnh@gmail.com (N.K.H.); khanhbq@fpt.vn (B.Q.K.); 3 Ha Nam Provincial General Hospital, Ha Nam 400000, Vietnam; hoangngochayhp@gmail.com; 4 Bach Mai Hospital, Hanoi 100000, Vietnam; nguyen.19742003@gmail.com; 5 Vietnam National Children’s Hospital, Hanoi 100000, Vietnam; dr.trandangxoay@gmail.com; 6 Department of Global Public Health, Karolinska Institutet, SE-171 77 Stockholm, Sweden; linus.olson@ki.se; 7 Training and Research Academic Collaboration (TRAC)—Sweden—Vietnam, Hanoi 100000, Vietnam; 8 Department of Women’s and Children’s Health, Karolinska Institutet, SE-171 77 Stockholm, Sweden

**Keywords:** fibrin monomer, systemic lupus erythematosus, pregnancy

## Abstract

Background: Pregnancies in patients with systemic lupus erythematosus (SLE) have always been considered high-risk. D-dimer is known for its role in excluding the diagnosis of thrombosis and has been associated with lupus reactivation; however, its physiological elevation during pregnancy limits its utility in this population. Fibrin monomer (FM) has been shown in multiple studies to remain stable in pregnant women. The objectives of this study were to evaluate D-dimer and FM levels, as well as to assess the role of FM in SLE activity during pregnancy. Methods: The subjects included 76 pregnant women with SLE diagnosed according to the Systemic Lupus International Collaborating Clinics (SLICC) 2012 criteria. The assessment of disease activity was in accordance with the Systemic Lupus Erythematosus Pregnancy Disease Activity Index (SLEPDAI)**.** Results: The log_10_-transformed D-dimer (LtDD) and FM (LtFM) concentrations in the pregnant women with lupus were 1.229 (0.722–1.953) and 4.17 (3.01–5.34) µg/mL, respectively. A multivariate regression indicated that 59.1% of the variation in LtDD was influenced by the gestational age and SLEPDAI, while only 18.3% of the fluctuation in LtFM was affected by these factors. The concentration of LtFM was an independent factor in predicting SLE flare and disease activity level according to the SLEPDAI in pregnant women. Conclusions: In conclusion, this study’s findings suggest that elevated levels of both D-dimer and FM were observed in pregnant patients with SLE. However, only FM levels can be used as a prognostic factor in assessing the risk of SLE reactivation during pregnancy.

## 1. Introduction

Systemic lupus erythematosus (SLE) is a systemic autoimmune disease, causing multi-organ damage with chronic progression. The disease occurs mainly in women, mostly those in the reproductive age group [1,2]. Pregnancy is considered a burden on the immune system in patients with SLE. Hormonal changes during pregnancy induce a shift in lymphocyte function, favoring Th2 predominance. In some Th2-mediated autoimmune diseases, such as SLE, a rise in Th2 can exacerbate disease activity and lead to adverse outcomes, which may contribute to the 20-fold higher maternal mortality rate compared to that of pregnant women without SLE [3,4]. Moreover, pregnancy in patients with SLE is associated with increased maternal and fetal complications compared to the general population. Common complications in pregnant women with SLE include disease flares, hypertension, preeclampsia, preterm labor, miscarriage, and thrombosis. Several previous studies have reported that the rate of disease flares during pregnancy may reach up to 68% [5,6]. The risk of lupus flares significantly increases in women with a history of lupus nephritis or those with new-onset lupus during pregnancy [7].

D-dimer is a known marker used to rule out thrombosis with high sensitivity and has a negative predictive value for ruling out deep vein thrombosis (DVT), but has a very low specificity and positive predictive value for pulmonary embolism (PE) [8]. The D-dimer concentration is influenced by a variety of physiological and pathological factors. According to Johnson et al. (2019), D-dimer levels increase with age, pregnancy, inflammation, infection, malignancy, trauma, and recent surgery [9]. These conditions can trigger coagulation and fibrinolysis, resulting in elevated D-dimer levels even in the absence of thrombosis. Additionally, liver dysfunction can impair D-dimer clearance, further contributing to elevated values. The high sensitivity but low specificity of the D-dimer assay underscores the importance of interpreting results in the clinical context. Therefore, elevated D-dimer levels should be cautiously evaluated, especially in populations with naturally elevated baseline levels, such as pregnant or elderly individuals. In SLE patients, D-dimer concentrations are higher than those in healthy people, but no thrombosis occurs, which is thought to be related to systemic infection and disease activity [10]. Another study demonstrated that D-dimer levels were positively correlated with the Systemic Lupus Erythematosus Disease Activity Index (SLEDAI), and were associated with factors such as anti-dsDNA antibodies, erythrocyte sedimentation rate (ESR), C-reactive protein (CRP), and complement components C3 and C4, as well as multi-organ involvement [11]. However, although D-dimer levels increase with gestational age, they have limited prognostic value in pregnancies at high risk for placenta-mediated complications. Therefore, D-dimer is not a suitable marker to either exclude thrombosis or assist in assessing the level of SLE activity in pregnant patients.

Fibrin monomer is formed from fibrinogen under the action of the enzyme thrombin during the final stage of the coagulation cascade. Specifically, thrombin (factor IIa) cleaves fibrinopeptides A and B from fibrinogen, resulting in the formation of fibrin monomers, which then spontaneously polymerize into a fibrin network. A mild increase in fibrin monomer concentration is considered a physiological finding during pregnancy due to the naturally occurring hypercoagulable state, particularly in the third trimester. However, according to Kristoffersen et al. (2019), fibrin monomer (FM) levels generally remain within the reference range throughout pregnancy and are more stable compared to other coagulation markers such as D-dimer [12]. Their study, which monitored FM concentrations in healthy pregnant and postpartum women, found that while FM levels were slightly elevated during pregnancy compared to non-pregnant controls, they did not show significant fluctuations across trimesters. In contrast, D-dimer levels rose progressively with gestational age. This stability suggests that FM may serve as a more reliable marker for detecting pathological coagulation changes during pregnancy, minimizing the confounding effects of physiological variations. Nevertheless, abnormally elevated fibrin monomer levels may indicate placental thrombotic–ischemic complications, such as preeclampsia, disseminated intravascular coagulation (DIC), or placental abruption. In pregnancies complicated by conditions such as deep vein thrombosis (DVT), fibrin monomer levels can rise significantly, highlighting its potential as an early marker of hypercoagulability [13]. Some studies in pregnant women have shown that FM could be a potential thrombotic prognostic marker with a higher specificity than that of D-dimer due to its fairly constant properties according to gestational age [12,13,14]. This has led us to assess concentrations of both markers during pregnancy and evaluate FM’s relationship with SLE disease activity in pregnancy.

## 2. Materials and Methods

Patients: This cross-sectional retrospective study included 76 pregnant SLE patients treated at the Centre of Allergy and Clinical Immunology and the SLE management unit of the Outpatient Department at Bach Mai Hospital from July 2020 to March 2021. Subjects were excluded if (1) they had other autoimmune diseases, (2) malignancy, (3) kidney failure, or (4) sepsis/septic shock, or if (5) they were taking anticoagulant drugs. Details were collected through medical records, including age, gestational age, disease duration, and clinical and laboratory manifestations such as skin lesions, mucocutaneous lesions, arthritis, alopecia, hematologic disorders, serositis, nephritis, anti-dsDNA antibodies, C3 and C4 complement, low complement, and SLE flare. Basic hematologic and coagulation indices were collected, including platelet number (PLT), prothrombin time (PT), activated partial thromboplastin time (APTT), fibrinogen concentration, and D-dimer and FM concentrations. Coagulation tests were performed and quality-controlled on the STA R Max^®^-Stago automated coagulation analyzer [15]. D-dimer and fibrin monomer (FM) concentrations were measured using latex-enhanced immunoturbidimetric assays on the STA R Max^®^ 2 automated coagulation analyzer (Diagnostica Stago, Asnières-sur-Seine, France) with compatible assay kits. All tests were performed in accordance with the manufacturer’s instructions and under quality control procedures.

Statistical analysis: Data were analyzed using the IBM SPSSv16.0 and R project version 4.1.0 programs, applying the *p*-value method with a statistical significance level of α = 0.05. (1) Categorical variables were described as number (n) and proportion (%), and χ^2^ test was used to compare proportions; (2) continuous variables were described as mean ± standard deviation and median (interquartile range, IQR) and were compared using the Mann–Whitney U test (between 2 variables) or Kruskal–Wallis test (when more than 2 variables). The correlation between two quantitative variables was evaluated based on Pearson’s correlation coefficient. The independent variables that affected the dependent variables were determined using univariate linear regression (for continuous outcome variables) or logistic regression (for binary outcome variables). The final model, which was also the optimal and most suitable multivariate regression model (with the fewest independent variables and highest predictive ability), was selected using the Bayesian Model Averaging (BMA) method in R (version 4.1.0). The D-dimer and FM concentrations were transformed into log10 to bring them to a normal or near-normal distribution. There were no missing data.

Applied standards: SLE diagnosis was made according to the disease classification system of the Systemic Lupus International Collaborating Clinics (SLICC) 2012 [16]. SLE activity during pregnancy was assessed according to the Systemic Lupus Erythematosus Pregnancy Disease Activity Index (SLEPDAI) [17]. The exacerbation of SLE during pregnancy was defined according to the definition given in the SELENA SLEDAI study by Buyon et al. [18]. Gestational age was grouped into 1st trimester (≤13^6/7^ weeks), 2nd trimester (14^0/7^–27^6/7^ weeks), and 3rd trimester (≥28^0/7^ weeks) [19]. Gestational age was estimated based on crown–rump length during fetal ultrasound. The normal threshold for D-dimer was <0.5 µg/mL, and for FM, it was <6.0 µg/mL, according to the manufacturer’s reference values.

Use of Generative AI Tools: Preparation for the manuscript included support from ChatGPT (OpenAI, GPT-4-turbo, last updated by OpenAI in April 2024) to assist in grammar correction and language improvements during manuscript writing. Scientific contents were generated independently by the authors.

## 3. Results

### 3.1. Characteristics of the Study Group

The average age of the group of pregnant women with SLE was 27.9 ± 4.7 years. The main clinical manifestations at the time of the study were nephritis in 36 cases (47.4%) and hematological disorders in 26 cases (34.2%). There were 25 pregnant women (32.8%) exhibiting SLE flare (Table 1).

### 3.2. Laboratory Indices of the Participants

**Figure 1:**
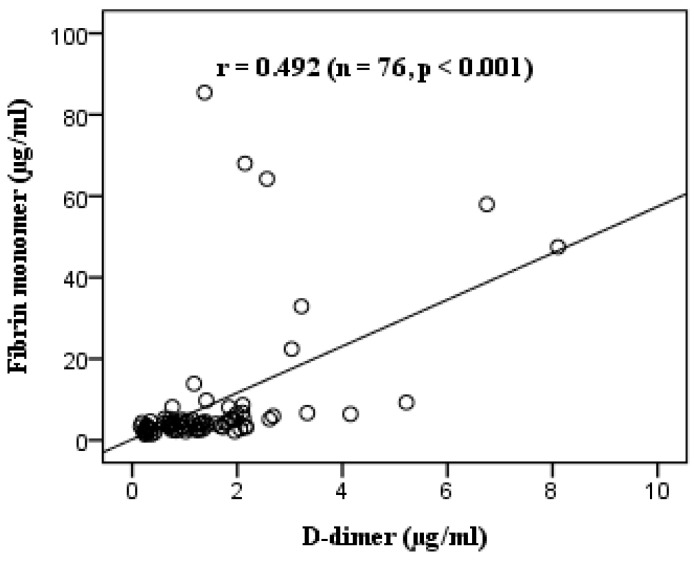
Correlation between D-dimer and FM in participants. r: Pearson’s correlation.

The median D-dimer and FM concentrations were 1.229 (0.722–1.953) µg/mL and 4.17 (3.01–5.34) µg/mL, respectively. Although the median FM level was within the normal range, 21.1% of patients exhibited FM concentrations that were more than twice the upper reference limit. Differences in D-dimer concentrations were observed across all trimesters, whereas differences in FM concentrations were noted only between the first and third trimesters (Table 2). The FM concentration was moderately correlated with the D-dimer concentration, with r = 0.492 (*p* < 0.001) (Figure 1).

### 3.3. Factors Affecting the D-Dimer and FM Concentration in Pregnant Patients with SLE

LtDD and ltFM were both positively correlated with the gestational age, anti-dsDNA antibody concentration, and SLEPDAI score (Table 3).

**Figure 2:**
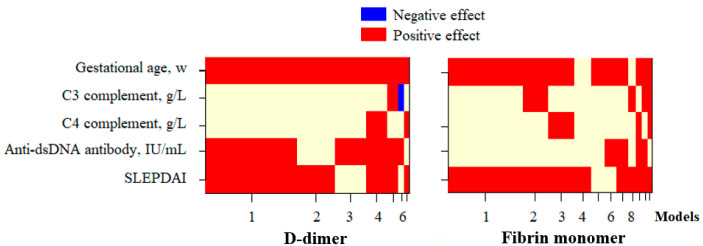
Multivariate regression models selected by the Bayesian Model Averaging method to examine variables affecting log_10_-transformed D-dimer and FM concentrations. Blue/red blocks indicate independent variables with negative/positive effects, respectively, on the dependent variable in each selected model; Beige blocks represent variables that were either not included in the corresponding model or had no statistically significant effect. Models: the rankings of selected models were based on posterior probability, calculated using the BMA method. D-dimer: Log10-transformed D-dimer; Fibrin monomer: Log10-transformed FM; Anti-dsDNA: anti-double-stranded deoxyribonucleic acid; SLEPDAI: SLE Pregnancy Disease Activity Index.

The multivariate regression analysis demonstrated that the most optimal model for predicting the fluctuation in ltDD and ltFM was based on the Bayesian Model Averaging (BMA) method, which incorporated the gestational age, anti-dsDNA antibody concentration, and SLEPDAI score (Figure 2). Accordingly, 59.1% of the fluctuation in ltDD was influenced by these factors, while only 18.3% of the variation in ltFM was affected by the gestational age and SLEPDAI score (Table 4).

### 3.4. Roles of D-Dimer and FM in Assessing SLE Disease Activity During Pregnancy

**Figure 3:**
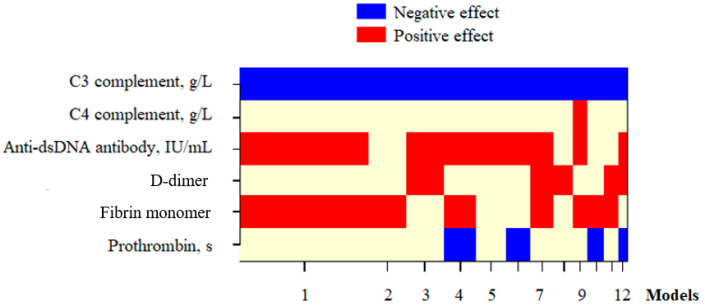
Multivariate regression models selected by the Bayesian Model Averaging method to examine variables affecting SLEPDAI. Blue/red blocks indicate independent variables with negative/positive effects, respectively, on the dependent variable in each selected model; Beige blocks represent variables that were either not included in the corresponding model or had no statistically significant effect. Models: the rankings of selected models were based on posterior probability, calculated using the BMA method. D-dimer: Log10-transformed D-dimer; Fibrin monomer: Log10-transformed FM; Anti-dsDNA: anti-double-stranded deoxyribonucleic acid; SLEPDAI: SLE Pregnancy Disease Activity Index.

The univariate regression analysis indicated that the concentrations of complement components C3 and C4, anti-dsDNA antibodies, ltDD, ltFM, and prothrombin had impacts on the SLEPDAI score (Table 5). However, it could be seen through the multivariate regression analysis that only ltFM, along with the complement component C3 and anti-dsDNA antibody concentrations, were significant factors in predicting the changes in disease activity according to the SLEPDAI score, with R^2^ = 0.551 (Figure 3, Table 5).

**Figure 4:**
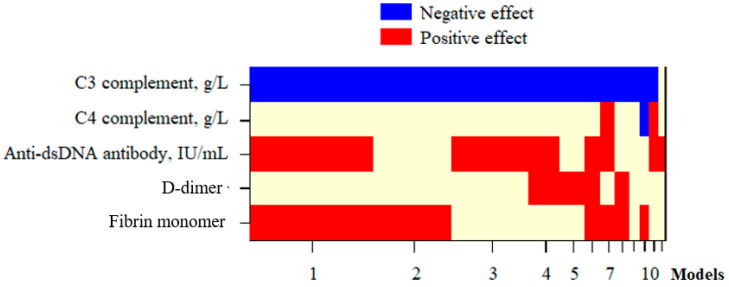
Multivariate regression models selected by the Bayesian Model Averaging method to examine variables affecting SLE flare. Blue/red blocks indicate independent variables with negative/positive effects, respectively, on the dependent variable in each selected model; Beige blocks represent variables that were either not included in the corresponding model or had no statistically significant effect. Models: the rankings of selected models were based on posterior probability, calculated using the BMA method. D-dimer: Log10-transformed D-dimer; Fibrin monomer: Log10-transformed FM; Anti-dsDNA: anti-double-stranded deoxyribonucleic acid; SLEPDAI: SLE Pregnancy Disease Activity Index.

Regarding the roles of the D-dimer and FM concentration in predicting SLE flares during pregnancy, the univariate regression analysis showed that complement components C3 and C4, anti-dsDNA antibody levels, ltDD, and ltFM had the ability to predict SLE flares during pregnancy (Table 6). However, multivariate regression analysis indicated that ltFM concentration was a significant factor in predicting the acute phase of SLE during pregnancy (OR: 6.177; 95% confidence interval: 1.259–30.308), along with complement component C3 levels and anti-dsDNA antibody concentrations (Figure 4, Table 6).

## 4. Discussion

In our study, pregnant patients with lupus had significantly higher average D-dimer concentrations than the new threshold value for healthy pregnant women suggested by some authors. The D-dimer concentration of pregnant women with lupus in all trimesters, in some cases, exceeded this reference threshold without embolism complications (Table 2) [20,21,22,23]. Several studies have demonstrated that the use of a higher cutoff D-dimer value to discriminate normal from abnormal results could compensate for the higher baseline D-dimer values during pregnancy and improve the specificity of sensitive D-dimer assays, without lowering their sensitivity [24,25,26]. Physiological changes during gestation, particularly the progressive activation of coagulation pathways, lead to a steady rise in baseline D-dimer levels, thereby limiting the specificity of standard thresholds used to rule out venous thromboembolism (VTE). Chan et al. (2010) suggested that the use of trimester-specific D-dimer cutoffs could help distinguish between physiological and pathological elevations, improving specificity without sacrificing sensitivity in suspected deep vein thrombosis (DVT) cases [24]. In line with this, van der Pol et al. (2019) proposed the Pregnancy-Adapted YEARS diagnostic algorithm, which incorporates clinical probability assessment and elevated D-dimer thresholds to safely exclude pulmonary embolism (PE), reducing the need for unnecessary imaging in pregnant women [25]. Additionally, Orita et al. (2021) identified optimal D-dimer cutoffs to exclude DVT and reported an association between elevated D-dimer levels and postpartum hemorrhage in cesarean section patients [26]. These findings support the rationale for using pregnancy-adjusted D-dimer reference ranges in both clinical practice and research settings. In our study, although D-dimer concentrations in pregnant patients with SLE were frequently above conventional thresholds, they did not consistently indicate thrombotic complications, highlighting the limitations of a fixed cutoff value in this population.

The median FM concentrations in pregnant women with lupus in our study were different between the first and third trimesters, with 21.1% participants developing FM levels that were more than twice the upper reference limit (Table 2). Several studies have consistently demonstrated that fibrin monomer (FM) concentrations remain relatively stable throughout gestation in healthy pregnant women, supporting its potential utility as a more specific thrombotic marker during pregnancy. In a study by Onishi et al., FM complex (FMC) levels were measured in 87 healthy pregnant women and compared to 127 non-pregnant women [13]. The results indicated that FMC concentrations remained largely unchanged during early and mid-pregnancy, with only a slight increase observed in late pregnancy. Importantly, FMC levels remained within the normal reference range across all stages of pregnancy and did not exceed the diagnostic threshold for deep vein thrombosis (DVT), even in the third trimester. This finding suggests that elevated FMC levels are uncommon in the absence of thrombotic or other pathological conditions during gestation. Similarly, Kawamura et al. evaluated the utility of FMC in screening for venous thromboembolism (VTE) in the late pregnancy and postpartum periods. Their study confirmed the stability of FMC concentrations and highlighted that sudden elevations were more likely to be associated with underlying complications rather than physiological changes in pregnancy alone [14]. Kristoffersen et al. further corroborated these findings in their longitudinal study, which measured FM concentrations in 20 healthy pregnant women and 19 non-pregnant controls [12]. The median FM levels were found to be slightly higher in pregnant women (6.2 mg/L) compared to non-pregnant controls (4.8 mg/L), yet these values remained stable throughout the three trimesters. Notably, the variations in FM concentrations across gestational stages were minimal, and no significant trimester-dependent increase was reported [12]. Taken together, these studies suggest that FM concentrations exhibit limited variability during healthy pregnancies and remain below clinically significant thresholds in most cases. This relative physiological stability enhances FM’s specificity as a biomarker when assessing thrombotic risk or other pregnancy-related complications. As such, deviations from this stable baseline in patient populations—such as in pregnant women with systemic lupus erythematosus (SLE)—may be more clinically meaningful and reflective of underlying disease processes rather than gestational physiology alone. As our study was retrospective in design, we did not include a control group of healthy pregnant women at comparable gestational ages. Therefore, comparisons were made based on previously established reference data from the aforementioned studies.

The correlation analysis showed that the D-dimer and FM concentrations displayed a moderate positive linear correlation (r = 0.492, *p* < 0.001) (Figure 1), which may be related to SLE activity during pregnancy, encouraging a search for factors influencing ltDD and ltFM in pregnant women with lupus. The correlation analysis demonstrated that the log_10_-transformed D-dimer and FM concentrations both have a weak linear correlation with the anti-dsDNA concentration and SLEPDAI score. Meanwhile, ltDD correlated moderately (r = 0.669) and ltFM correlated weakly (r = 0.310) with the gestational age (Table 3). Multivariate regression analysis demonstrated that gestational age, anti-dsDNA concentration, and SLEPDAI score significantly influenced D-dimer levels, collectively accounting for the majority of the variation in log-transformed D-dimer concentrations. In contrast, only a modest proportion of the variability in ltFM concentrations could be explained by gestational age and SLEPDAI score, indicating a weaker association (Figure 2, Table 4). In addition, several studies have investigated the role of D-dimer in lupus reactivation. Ferreira et al. (2019) showed that an SLEDAI-2K score >4 and D-dimer concentrations are closely related to SLE disease activity. In the group of patients with SLE, the D-dimer levels showed a positive correlation with the SLEDAI-2K score (r = 0.347, *p* = 0.003) [27]. Oh et al. demonstrated that the D-dimer test’s specificity was significantly lower in the SLE patients compared to that in the controls (28.4% vs. 84.4%) and was related to the SLE disease activity [28]. By contrast, no studies have yet explored the role of FM in this field.

Our study provided results aiming to fill this research gap. Among 76 participants, the log_10_-transformed D-dimer and FM concentrations did not correlate with the complement component C3 and C4 levels (Table 3). However, the multivariate regression analysis showed that ltFM, along with the complement component C3 and anti-dsDNA antibody concentrations, were independent factors affecting the SLEPDAI score (Figure 3, Table 5). Furthermore, ltFM and these indices were also capable of predicting SLE flares in the participants, with the probability (P) of SLE flare calculated using the logistic regression formula:

P = 1/(1 + e^−^ᶻ),

where z = 1.821 × ltFM + 0.012 × anti-dsDNA − 3.247 × C3 − 0.089 (Figure 4, Table 6). This illustrated the influence of indirect interactions between the complement system and the coagulation cascade on the SLE activity during pregnancy, as determined in SLE patients by Liang et al. [29]. The interplay between the complement system and the coagulation cascade plays a crucial role in the pathophysiology of systemic lupus erythematosus (SLE), especially during pregnancy when both systems are physiologically activated. As outlined by Liang et al., the complement and coagulation systems interact indirectly in a mutually reinforcing manner: complement activation can trigger thrombin generation, and coagulation proteases like thrombin can, in turn, amplify complement activation by cleaving C3 and C5. This bidirectional crosstalk contributes to a proinflammatory and prothrombotic state that may drive lupus activity and worsen clinical outcomes during pregnancy. In our study, we found that the log10-transformed fibrin monomer (ltFM) concentration, along with complement component C3 and anti-dsDNA antibody levels, were independent predictors of disease activity, as measured by the SLEPDAI score. This finding supports the hypothesis that fibrin monomer not only reflects a hypercoagulable state but also correlates with immunologic activity. The involvement of complement component C3 in this model further underscores the role of the complement–coagulation axis in driving lupus flares. These results align with mechanistic insights from previous studies and suggest that FM may serve as a sensitive integrated biomarker of both immunologic and thrombotic activation in pregnant women with SLE, warranting further longitudinal validation.

Nevertheless, there were some limitations of our study. Firstly, all of the blood samples and clinical data were taken from pregnant women with lupus receiving treatment. Secondly, we designed the study as a cross-sectional retrospective study. We did not conduct a longitudinal follow-up to assess the thrombotic complications, because at-risk pregnant women were administered anticoagulants as soon as this high risk was identified.

## 5. Conclusions

To conclude, the findings of our study suggest that elevated levels of both D-dimer and FM were observed in pregnant patients with SLE. While D-dimer was strongly influenced by various pregnancy-related factors, it showed a weak correlation with SLE disease activity. In contrast, the FM concentration appeared to be less affected by physiological changes during pregnancy and was identified as an independent predictor of disease flare and activity level, as assessed by the SLEPDAI score. Determining the definitive role of FM in predicting SLE reactivation will require future longitudinal follow-up studies in this population.

## Figures and Tables

**Table 1:** Demographic and clinical characteristics of participants.

Characteristics	Value
Age, y, mean ± SD median (IQR)	27.9 ± 4.7 27.5 (25.0–32.0)
Gestational age, w, mean ± SD median (IQR)	21.0 ± 10.3 22.0 (10.25–30.0)
Trimesters, n (%)	
1st	23 (30.3)
2nd	30 (39.4)
3rd	23 (30.3)
Disease duration, y, mean ± SD median (IQR)	6.3 ± 4.2 6.0 (3.0–9.75)
Clinical and laboratory manifestations, n (%)	
Skin lesions, n (%)	7 (9.2)
Mucocutaneous lesions, n (%)	3 (3.9)
Arthritis, n (%)	7 (9.2)
Alopecia, n (%)	8 (10.5)
Hematologic disorders, n (%)	26 (34.2)
Serositis, n (%)	6 (7.9)
Nephritis, n (%)	36 (47.4)
SLEPDAI, mean ± SD median (IQR)	5.5 ± 5.4 4.0 (1.3–9.0)
SLE flare, n (%)	25 (32.8)

SLE: systemic lupus erythematosus; SLEPDAI: SLE Pregnancy Disease Activity Index; SD: standard deviation; IQR: interquartile range.

**Table 2:** Laboratory indices in pregnant women with systemic lupus erythematosus.

Laboratory Indices	Value	1st Trimester (n = 23)	2nd Trimester (n = 30)	3rd Trimester (n = 23)	*p* *	P1-2 ^†^	P2-3 ^†^	P1-3 ^†^
C3 complement, g/L Median (IQR)	0.96 (0.70–1.21)	0.82 (0.69–1.02)	0.98 (0.61–1.24)	1.07 (0.91–1.22)	0.311	0.478	0.667	0.072
C4 complement, g/L Median (IQR)	0.17 (0.12–0.28)	0.21 (0.13–0.36)	0.13 (0.09–0.23)	0.18 (0.12–0.27)	0.062	0.022	0.161	0.373
Anti-dsDNA antibody, IU/mL Median (IQR)	33.8 (10.0–149.9)	33.5 (10.0–150.0)	34.0 (15.5–111.5)	77.7 (10.0–150.0)	0.921	0.743	0.876	0.723
Prothrombin, s Median (IQR)	11.0 (10.1–11.6)	11.5 (10.8–11.9)	10.8 (10.2–11.4)	11.0 (10.1–11.6)	0.074	0.030	0.886	0.078
APTT, s Median (IQR)	29.4 (27.4–30.8)	30.3 (28.9–34.8)	28.9 (26.7–30.4)	29.1 (26.0–30.8)	0.001	0.298	0.001	0.001
Fibrinogen, g/L Median (IQR)	4.26 (3.56–4.96)	3.83 (2.97–4.38)	4.04 (3.60–4.57)	4.94 (4.37–5.78)	0.032	0.009	0.733	0.068
Platelet, ×10^9^/L Median (IQR)	244 (189–280)	246 (156–298)	242 (193.5–283.7)	230 (186–270)	0.908	0.900	0.596	0.930
D-dimer, µg/mL Median (IQR)	1.229 (0.722–1.953)	0.370 (0.262–0.954)	1.198 (0.762–1.952)	1.840 (1.324–2.146)	<0.001	<0.001	0.006	<0.001
FM, µg/mL Median (IQR)	4.17 (3.01–5.34)	3.61 (2.33–4.76)	3.98 (2.76–6.08)	5.03 (3.38–8.69)	0.048	0.206	0.161	0.016
FM > 2 × Ref, %	21.1%				-	-	-	-

Continuous variables were described by median (interquartile range, IQR); * comparison among 3 groups according to Kruskal–Wallis test; ^†^ comparison between groups according to Mann–Whitney U test; Ref: reference limit.

**Table 3:** Correlation between log_10_-transformed D-dimer and FM concentration and gestational age and SLE disease activity during pregnancy.

Parameter	Log_10_-Transformed D-Dimer		Log_10_-Transformed FM	
	r	*p*-Value	r	*p*-Value
Gestational age, w	0.669	<0.001	0.310	0.006
C3 complement, g/L	−0.186	0.107	−0.054	0.644
C4 complement, g/L	−0.218	0.058	−0.020	0.867
Anti-dsDNA antibody, IU/mL	0.389	0.001	0.246	0.032
SLEPDAI	0.378	0.001	0.317	0.005

anti-dsDNA: anti-double-stranded deoxyribonucleic acid; SLEPDAI: SLE Pregnancy Disease Activity Index; r: Pearson’s correlation.

**Table 4:** Multivariate regression analysis to examine variables affecting log_10_-transformed D-dimer and FM concentrations.

Variable	Log_10_-Transformed D-Dimer		Log_10_-Transformed FM	
	Estimate (95%CI)	*p*-Value	Estimate (95%CI)	*p*-Value
Gestational age, w	0.023 (0.017–0.028)	<0.001	0.011 (0.003–0.018)	0.008
SLEPDAI	0.015 (0.003–0.027)	0.013	0.021 (0.006–0.035)	0.007
Anti-dsDNA antibody, IU/mL	0.001 (0.000–0.002)	0.016	-	-
R^2^	0.591	<0.001	0.183	0.001

anti-dsDNA: anti-double-stranded deoxyribonucleic acid; SLEPDAI: SLE pregnancy disease activity index; CI: confidence interval.

**Table 5:** Univariate and multivariate regression analyses to examine variables affecting SLEPDAI.

Variable	Univariate Analysis			Multivariate Analysis		
	Estimate	95%CI	*p*-Value	Estimate	95%CI	*p*-Value
Gestational age, w	0.039	−0.083–0.160	0.529			
C3 complement, g/L	−9.665	(−12.503)–(−6.826)	<0.001	−7.763	(−10.729)–(−5.080)	<0.001
C4 complement, g/L	−14.752	(−24.768)–(−4.737)	0.004			
Anti-dsDNA antibody, IU/mL	0.047	0.028–0.065	<0.001	0.022	0.005–0.039	0.012
LtDD	5.586	2.419–8.753	0.001			
LtFM	4.568	1.407–7.729	0.005	3.319	0.875–5.764	0.009
Prothrombin, s	−1.521	(−2.903)–(−0.139)	0.031			
APTT, s	−0.056	(−0.283)–0.172	0.628			
Fibrinogen, g/L	0.025	(−1.023)–1.073	0.962			
Platelet, ×10^9^/L	−0.014	(−0.030)–0.002	0.094			
R^2^	-		-	0.551		<0.001

anti-dsDNA: anti-double-stranded deoxyribonucleic acid; SLEPDAI, SLE Pregnancy Disease Activity Index; ltDD: log_10_-transformed D-dimer; ltFM: log_10_-transformed fibrin monomer; APTT: activated partial thromboplastin time; CI: confidence interval.

**Table 6:** Univariate and multivariate regression analyses to examine variables affecting SLE flare.

Variable	Univariate Analysis			Multivariate Analysis		
	Estimate	OR (95%CI)	*p*-Value	Estimate	OR (95%CI)	*p*-Value
Gestational age, w	0.002	1.002 (0.956–1.050)	0.924			
C3 complement, g/L	−3.601	0.027 (0.004–0.189)	<0.001	−3.247	0.039 (0.004–0.340)	0.003
C4 complement, g/L	−6.190	0.002 (0.000–0.358)	0.019			
Anti-dsDNA antibody, IU/mL	0.018	1.019 (1.009–1.028)	<0.001	0.012	1.012 (1.002–1.023)	0.024
LtDD	2.160	8.670 (1.713–43.876)	0.009			
LtFM	1.823	6.192 (1.481–25.880)	0.012	1.821	6.177 (1.259–30.308)	0.025
Prothrombin, s	−0.583	0.558 (0.311–1.001)	0.051			
APTT, s	−0.012	0.988 (0.906–1.077)	0.778			
Fibrinogen, g/L	0.069	1.071 (0.718–1.599)	0.736			
Platelet, ×10^9^/L	−0.002	0.998 (0.992–1.004)	0.537			

anti-dsDNA: anti-double-stranded deoxyribonucleic acid; ltDD: log_10_-transformed D-dimer; ltFM:log_10_-transformed fibrin monomer; APTT: activated partial thromboplastin time; OR: odds ratio; CI: confidence interval.

## Data Availability

The datasets presented in this article are not readily available because of ethical considerations and approval. Requests to access the datasets should be directed to the corresponding author.
